# Characterizing Online Questions and Answers About Cannabis Use and Pregnancy on the Social Media Platform Quora: Data Mining and Qualitative Content Analysis

**DOI:** 10.2196/82300

**Published:** 2026-08-14

**Authors:** Qing Xu, Christine Wenzel, Jiawei Li, Zhuoran Li, Mingxiang Cai, Cassandra L Taylor, Beverly J Wolpert, Catharine Trice, Erin South, Susan Bersoff-Matcha, Tim Mackey

**Affiliations:** 1 S-3 Research LLC, 9150 Chesapeake Drive, Suite 290, San Diego, CA, 92123, United States, 1 9514914161; 2 Global Health Policy and Data Institute, San Diego, CA, United States; 3 Center for Drug Evaluation and Research, U.S. Food and Drug Administration, Silver Spring, MD, United States; 4 Human Foods Program, Office of Surveillance Strategy and Risk Prioritization, U.S. Food and Drug Administration, College Park, MD, United States; 5 Office of Women’s Health, U.S. Food and Drug Administration, Silver Spring, MD, United States; 6 Department of Anthropology, University of California, San Diego, San Diego, CA, United States; 7 San Diego Supercomputer Center, San Diego, CA, United States

**Keywords:** cannabis, pregnancy, maternal health, Quora, women health

## Abstract

**Background:**

This study leveraged data mining from the question-and-answer social media platform Quora to explore attitudes and experiences concerning cannabis use and pregnancy.

**Objective:**

The primary aim was to identify and characterize online discourse specific to risk perceptions associated with cannabis use and pregnancy.

**Methods:**

Data collection encompassed two layers of Quora data: user-generated questions and answers to these questions. Keywords related to cannabis and pregnancy guided data selection and collection. Manual content coding was used for text analysis, with coding conducted by multiple coders, and interrater reliability assessed. Publicly available user profile metadata was reviewed to understand the roles of contributors participating in online discussions. A chi-square test was used to examine potential associations between sentiment toward cannabis use during pregnancy and sentiment toward general cannabis use.

**Results:**

We collected 7834 questions pertaining to cannabis use, with 6.8% (n=529) identified as relevant to topics about pregnancy. Questions spanned from January 2012-July 2023. Content analysis classified the detected themes into two stages of discussion: (1) cannabis use before pregnancy and (2) cannabis use during pregnancy, and seven subthemes: (a) safety of cannabis use (608/1074, 56.6%), (b) removal of cannabis from the body (372/1074, 34.6%), (c) legal consequences of cannabis use (175/1074, 16.3%), (d) cannabis use history before the pregnancy (27/529, 5.1%), (e) purpose of cannabis use (22/529, 4.2%), (f) public opinion comments (21/529, 4.0%), and (g) dosing recommendations (2/529, 0.4%). Among 1916 answers to these questions, 56.1% (n=1074) were relevant to cannabis and pregnancy topics. Among all answer contributors, 78.0% (660/846) were nonexpert observers (ie, users who did not publicly disclose professional credentials or affiliations on their Quora profiles), 6.3% (53/846) were people who had used cannabis during pregnancy, 11.3% (96/846) were expert observers (ie, health care providers, researchers), and 4.4% (37/846) were cannabis industry observers. Questions related to removing cannabis from the body attracted the highest engagement. Notably, analysis of answers found predominantly negative sentiment among Quora users regarding cannabis use during pregnancy.

**Conclusion:**

This study identified an active online community discussing cannabis and pregnancy on Quora. Results highlight a diversity of discussion topics, high frequency of nonexpert observers providing advice, and generally an overall negative sentiment toward cannabis use during pregnancy but not necessarily toward general cannabis use not associated with pregnancy. Additional research is needed to understand information gaps and misinformation in cannabis and pregnancy safety discussions online and how to conduct effective outreach to pregnant women and their families.

## Introduction

Recent analysis of data from the National Survey on Drug Use and Health from 2002‐2020 has identified increasing use of cannabis (derived from the plant *Cannabis sativa* L) or various cannabis-derived products among pregnant women in the United States [1,2]. Cannabis use during pregnancy has increased significantly, rising by an estimated 64% from 2001 to 2014 [1]. Another recent study identified a significant increase in cannabis use among pregnant women in Northern California during the COVID-19 pandemic [3]. Despite increases in use, a substantial research gap persists regarding the impact of maternal cannabis use on child developmental outcomes [4]. Further, there are other social and legal implications associated with cannabis use for women who are pregnant, in part due to differences in state-level legal status of medical and adult use cannabis, as well as the current controlled status of marijuana at the federal level. With the rising prevalence of cannabis use during pregnancy and increased access accompanied by the introduction of new and emerging cannabis-derived products, understanding the health implications for both the mother and the developing fetus has become an important public health challenge.

Cannabis contains a class of compounds known as cannabinoids, which includes cannabidiolic acid and delta-9 (Δ^9^)-tetrahydrocannabinolic acid. When heated, such as during smoking or baking, cannabidiolic acid and delta-9 (Δ^9^)-tetrahydrocannabinolic acid can undergo nonenzymatic decarboxylation to form cannabidiol and Δ^9^-tetrahydrocannabinol (THC), respectively [5]. THC interacts with the mammalian endocannabinoid system, which plays an important role in fetal development and physiological regulation. During pregnancy, the mammalian endocannabinoid system becomes particularly critical for fetal brain development [6,7], and the presence of THC may pose developmental challenges for the fetus [8,9]. Recent research has shown that THC can traverse the placenta to the fetus, warranting further investigation into the implications of the passage of THC through the placenta [4]. Animal studies indicate that behavioral changes in offspring, such as heightened anxiety and drug-seeking tendencies, are associated with prenatal cannabis exposure [10]. In humans, maternal cannabis use has been linked to adverse cognitive and mental health outcomes in children, including autism, depression, and psychosis [11-14]. Observational studies have found associations between cannabis use and preterm birth, as well as low birth weight [15]. Further, the US Surgeon General issued an advisory on marijuana use during pregnancy and its potential risks, echoing concerns from the American College of Obstetricians and Gynecologists and a similar Food and Drug Administration consumer update on cannabis safety during pregnancy [16,17].

Prior research indicates that attitudes and perceptions toward cannabis use are important determinants of use during pregnancy. For example, one study found that women who continued to use cannabis prenatally were less likely to believe that cannabis could cause harm during pregnancy compared to women who quit, while another study found an increasing percentage of pregnant women perceive that regular cannabis use has no risk [18,19]. Studies have shown that legalization and increased access may further shape perceptions by reducing stigma and normalizing use [19]. However, evidence also suggests that pregnant women often encounter inconsistent or unclear information, particularly from informal sources, which may influence decision-making [20]. This may be in part due to the changing cannabis legalization landscape in the United States, beginning with legalization of adult use (ie, recreational use) first in Colorado and Washington in 2012, subsequent expansion to other states, efforts to decriminalize possession, the Agriculture Improvement Act of 2018 that removed hemp from the Controlled Substances Act, the recent changes to the definition of hemp in the Continuing Appropriations, Agriculture, Legislative Branch [21], and ongoing policies aimed at rescheduling or descheduling marijuana from the Controlled Substances Act [22,23]. These changes have been associated with increased availability, evolving public perceptions, and the introduction of public health guidance regarding cannabis use during pregnancy [18]. Such shifts may influence how individuals perceive risks and seek information, providing important context for trends observed in online discussions regarding cannabis use.

Research leveraging infodemiology (ie, studying the determinants and distribution of information in electronic media to inform public health) can help uncover insights that may not be available through traditional qualitative or survey-based methods, particularly for behaviors that are stigmatized (eg, marijuana use remains illegal at the US federal level and in some states). Using infodemiology approaches, several studies have specifically examined social media data to characterize cannabis use behavior, changes in the cannabis product and retail marketplace, and discussions about cannabis safety [24-27]. However, only a few studies to our knowledge have attempted to analyze social media data for cannabis-related topics specific to pregnancy [28,29].

Quora was launched in 2010 and has an estimated 400 million monthly users made up of approximately 37% US accounts [30,31]. Users may post questions, answer questions, comment on answers, and interact with content by following, sharing, and upvoting other users’ answers. The platform also helps users identify relevant content for health information seeking, including providing links to related questions, interaction metrics that encourage high-quality answers, and the ability to follow specific topics [32]. Additionally, each Quora user maintains a profile where they can voluntarily share demographic information, educational background, and work experience. Compared to other social media platforms (eg, X [formerly Twitter] [X Corp], Reddit [Reddit Inc], Facebook, Instagram [Meta Platforms, Inc]), Quora is relatively underutilized in infodemiology-related research, with only approximately 20 published studies that have examined Quora for discussions about addiction recovery, nicotine vaping, COVID-19, cancer, mental health, safe food handling, and health equity challenges, but none specifically examining cannabis and pregnancy-related topics [33-40]. Hence, this study provides an analysis of Quora question and answer content specific to cannabis use during pregnancy.

## Methods

**Figure 1.**
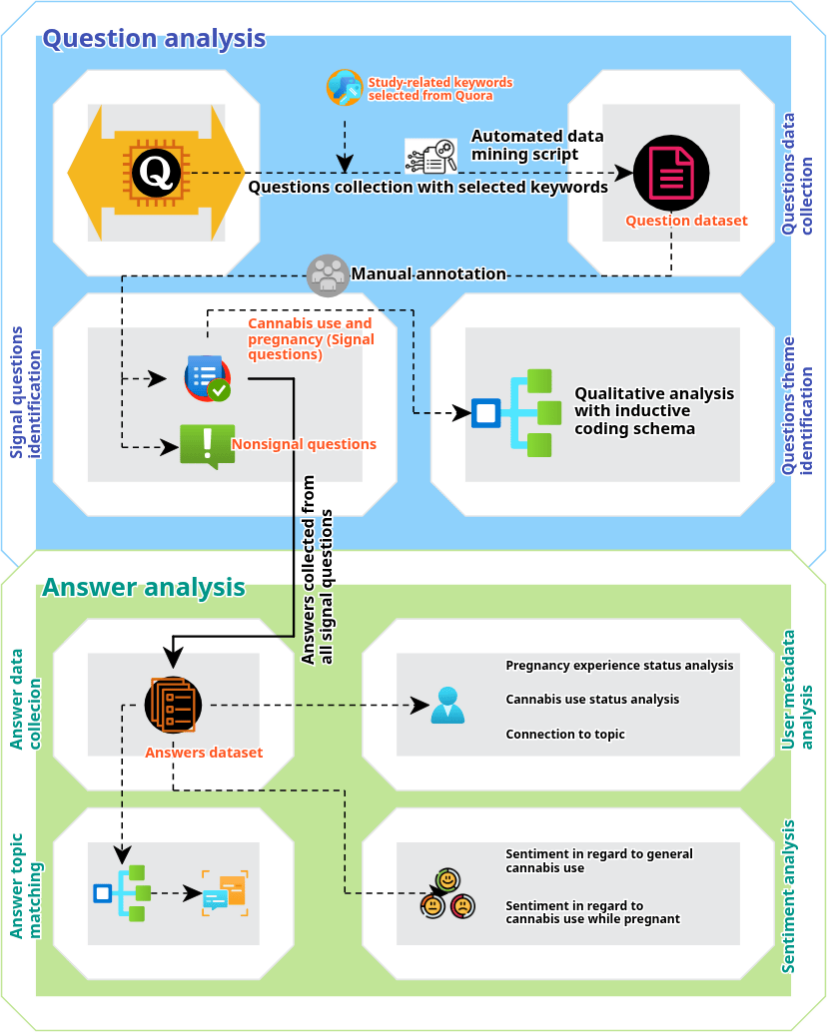
Summary of study methodology including data collection, data analysis, and content coding for Quora questions and answers.

This study was conducted in two phases: (1) data mining to collect question and answer data related to cannabis and pregnancy from the Quora platform and (2) content analysis of questions and answers using an inductive and deductive coding approach to characterize specific question themes and sentiment and interaction metrics associated with answers to questions. A visual summary of the study methodology is provided in Figure 1.

### Data Collection

In the absence of publicly available datasets or an official Quora API, we developed an automated data mining programming script to collect user-generated data from Quora. To collect data, we used a custom web scraping tool to extract all publicly available Quora questions and related answers, as well as associated metadata for users who answered questions. We also extracted related questions recommended by the platform when conducting automated data mining to collect questions. The web scraping tool used in this study was created in Python (Version 3.7.3, Python Software Foundation) with the Beautiful Soup library package (Version 4.9.3; Leonard Richardson). This data was stored in a JSON file and converted to an Excel file (Microsoft Corporation) for content analysis. We performed data collection from August to September 2023. Quora content posted during the time period of data collection was included as was any content available prior to this time period. All analyzed data were collected from publicly accessible Quora content available at the time of data collection.

To collect posts with discussion about cannabis and pregnancy topics, we first generated two lists of cannabis and pregnancy keywords by manually searching for questions on the Quora platform. We started with MeSH “cannabis” and “pregnancy” terms as our baseline general search terms and searched these two sets of keywords on Quora’s platform using the platform’s built-in search query function. This query function enabled us to collect additional keywords associated with cannabis and pregnancy experiences observed from the first 50 returned questions from each initial search term used. This step enabled us to generate from Quora online discussions a more comprehensive list of cannabis and pregnancy-related experience keywords (see Table 1 for the full list of study keywords) that was then used for additional structured searches and data collection. Data for Quora questions included textual content and the number of answers for each question.

**Table 1.** Study list of cannabis and pregnancy-related keywords.

	Cannabis associated keywords	Pregnancy associated keywords
MeSH terms	Cannabis, Hemp plant, Hemp plants, Hemp, Hemps, Plants, Marihuana, Marijuana, Cannabis indica, Cannabis sativa, Hashish, Hashishs, hang, Bhangs, Ganja, and Ganjas	Pregnancies, Gestation
Keywords selected from Quora	THC^a^, CBD^b^, Weed, DAB^c^, Marijuana, Pod, Blunt, Joint, and Edible	Birth, Miscarriage, Conceive, Pregnant, Placenta, Fetus, Unborn, and Baby

a THC: Δ9-tetrahydrocannabinol.

b CBD: cannabidiol.

c DAB refers to “dabbing,” a method of consuming cannabis concentrates.

After identifying relevant questions on cannabis and pregnancy topics, we used the same automated data mining script to collect associated user-generated answers confirmed by manual annotation as relevant to the study objective (ie, signal) that were then further analyzed. Our data mining tool collected the textual content of all underlying answers associated with a relevant question, publicly available user profile information for those who answered the relevant question, upvote numbers, and answer postdates.

### Content Analysis

Content analysis consisted of two different manual annotation methods to identify relevant thematic topics for Quora questions, including an inductive coding approach, and manual annotation and characterization of type of answer content, general sentiment toward cannabis use, and information about the types of users posting answers using a deductive coding approach. Additional analyses included assessing user engagement and interaction on questions and answers.

#### Question Content Analysis

Our analysis of Quora questions focused on identifying themes associated with knowledge, experiences, attitudes, and specific concerns regarding cannabis use before and during pregnancy. Two coders (QX and CW) independently used a binary coding approach for categorizing all collected questions as relevant or nonrelevant to the study objectives. We specifically excluded questions that were not relevant to the topic of cannabis use and pregnancy. Unrelated questions, such as those concerning tobacco use during pregnancy (ie, not cannabis use), family relations during pregnancy (eg, questions related to divorce proceedings regarding nonpregnant family members using cannabis products), and non–pregnancy-related cannabis use, were omitted from further analyses. However, questions mentioning “smoking” without specifying cannabis-related or tobacco-related topics were retained to collect respective answers and assess if they were related to cannabis themes. We used a general inductive coding approach to characterize specific themes of questions. The first author (QX) reviewed all questions, noting general themes from which the initial code list was formulated. Formal coding of the textual data followed, with codes refined and subcodes developed. The final coded dataset underwent review by the second author (CW). Any disparities in code definitions or applications between the first (QX) and second (CW) authors were resolved through consensus discussions, ensuring consistent coding across the dataset. Interrater reliability was assessed using Cohen kappa statistic. Considering a single question may cover multiple topics, each question could be coded with multiple theme labels. Additionally, descriptive statistics were used to analyze question characteristics, topic distribution, and volume over time.

#### Answer Content Analysis

For the Quora answers analysis, we initially used a binary coding approach to categorize all collected responses as either pertinent or irrelevant to the research objectives, aligning with the inclusion and exclusion criteria used for question analysis. However, responses to questions mentioning smoking without specifying cannabis-related or tobacco-related products were treated as non-signal if neither the question nor the answers indicated a specific product or substance. Subsequently, we implemented a deductive coding schema, focusing on three key aspects of user-generated interactions, including identifying the specific content type of answers, sentiment toward general cannabis use, and general sentiment regarding cannabis use during pregnancy. The specific content types of answers included (1) sharing personal anecdotes (eg, self-reported experiences or observed experiences), (2) expressing general opinions about cannabis use and pregnancy, (3) discussing research on cannabis and/or pregnancy, and (4) addressing laws, federal regulations, or recommendations concerning cannabis and pregnancy or other related issues. The sentiment coding schema for both general cannabis use and use during pregnancy consisted of the following categories: (a) positive sentiment, (b) neutral sentiment, (c) negative sentiment, and (d) unknown or not discussed. Sentiment classification was conducted manually, with positive sentiment reflecting supportive attitudes toward cannabis use, negative sentiment reflecting discouraging views or concerns regarding use, neutral sentiment reflecting informational or nonopinionated responses, and unknown indicating that sentiment was not clearly expressed. Two coders (QX and CW) independently reviewed and coded all answers, and any coding disagreements were resolved through consensus discussion. Additionally, a chi-square test was performed to examine the potential significance of the relationship between cannabis use sentiment during pregnancy and general cannabis use sentiment reported by users. We also applied a continuity correction as frequencies of zero were observed in certain categories. A *P* value of <.05 was considered statistically significant.

#### Topic Interaction Analysis

To better characterize the popularity or levels of user engagement for various question and answer topics regarding the use of cannabis during pregnancy, we explored users’ engagement patterns with questions and answers analyzed. The general popularity and level of interaction of questions was assessed based on the total number of answers a question received, while the answers were gauged by assessing the number of upvotes received.

#### User Metadata Analysis

To better characterize the types of Quora users who actively engaged in questions and answers concerning cannabis use and pregnancy, we analyzed publicly available metadata of Quora profiles for users who posted answers that were classified as relevant (ie, signal). We classified users’ status based only on publicly available self-reported profile information from textual data. Educational and working experience information from the user’s Quora profile or “Credentials & Highlights” section was also collected and evaluated in this analysis. The following categories were applied to classify user characteristics: (1) experience with pregnancy, including whether they were pregnant when they posted the answer, formerly pregnant, or had no self-reported information regarding pregnancy status; (2) current cannabis use, including if they were self-reported cannabis users or self-reported nonusers, or if they had unknown cannabis use; and (3) general professional affiliation or background, including if they self-reported as an expert (eg, health care providers, medical researchers, social work professionals, or similar professionals), industry actors (members of the cannabis industry), nonexpert observers (users who did not self-disclose professional credentials or affiliation), or women with direct experience with the topic (ie, those who have used cannabis while pregnant). Data was collected for purposes of aggregation, and no results contained in this study include individually identifiable information.

### Ethical Considerations

This study was reviewed by the WCG Institutional Review Board (WCG IRB) and determined to be exempt from Institutional Review Board oversight under 45 CFR §46.104(d)(4) because it involved the analysis of publicly available information. The study did not involve direct interaction or intervention with human participants; therefore, informed consent was not required. Only publicly available user-generated content and self-disclosed profile information from Quora were collected and analyzed. Data were aggregated and deidentified for analysis and reporting, and no individually identifiable information is presented in this manuscript.

## Results

### Overview

Our data collection yielded a total of 7834 questions from Quora, including 835 questions generated from direct search results, and 6999 collected as related questions referred by the Quora platform. After applying our binary coding approach, we identified 6.8% (529/7834) questions related to cannabis use and pregnancy, with the oldest question posted in January 2012 and the most recent posted in July 2023. Of the 1916 answers collected from selected relevant signal questions, 1074 (56.1%) answers were identified as related to cannabis use and pregnancy. Of the answers with readable timestamps, the earliest and latest dates were September 2012 and August 2023, respectively.

### Question Analysis

**Figure 2.**
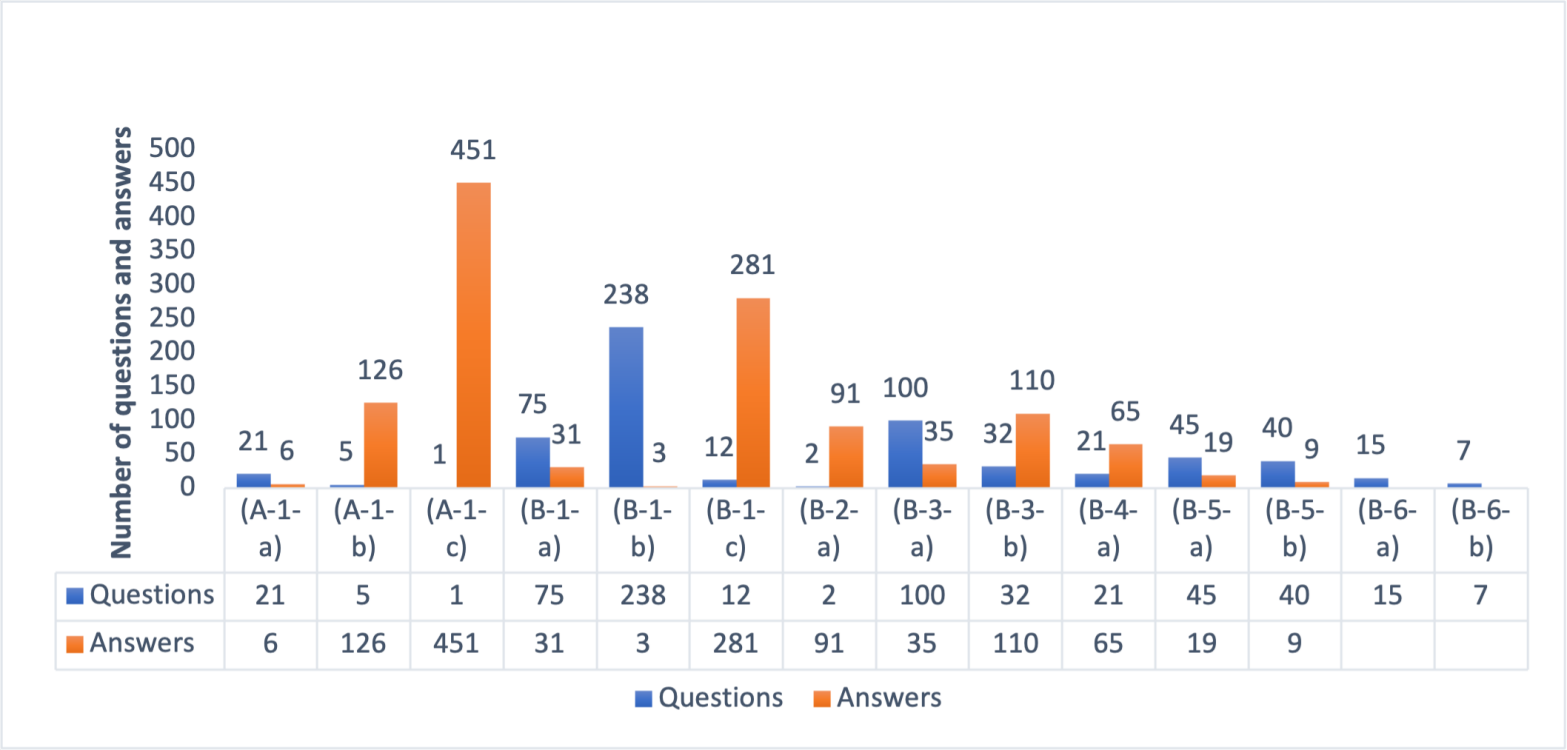
Counts of quora questions and corresponding answers by topic (Themes A-B). Topic codes and descriptions: A-1-a: impact of cannabis use on fertility; A-1-b: impact of prepregnancy cannabis use on pregnancy and fetus; A-1-c: recommended time between stopping cannabis use and conceiving; B-1-a: safety during pregnancy; B-1-b: safety of fetus; B-1-c: withdraw cannabis use for safety consideration; B-2-a: dosing recommendations; B-3-a: time period needed to remove cannabis from the body; B-3-b: method of removal of cannabis from the body before delivery; B-4-a: public opinion on using cannabis while pregnant; B-5-a: consequences of using cannabis while pregnant; B-5-b: consequences of testing positive while pregnant; B-6-a: self-reported therapeutic cannabis use; B-6-b: adult cannabis use.

Using an inductive coding schema, our qualitative analysis identified 14 question topics categorized under two major parent domains with some questions including both domains: cannabis use before pregnancy (27/529, 5.1%) and cannabis use during pregnancy (503/529, 95.1%). These were further subdivided into seven subcodes. (see Table 2 along with deidentified examples of questions for each subcode and Figure 2 for a breakdown of content classification volume). All questions were reviewed by the first (QX) and second author (CW), coded independently, and demonstrated high intercoder reliability for Quora question classification (Cohen κ=0.93).

**Table 2.** Summary statistics of quora question (n=529) and corresponding answer topics (n=1074).

Parent code	Description	Code	Description	Question titles	Questions (% of total signal questions n=529), n (%)	Answers (% of total signal answers, n=1074), n (%)
Cannabis use before pregnancy						
(A-1)	User’s history of cannabis use	(A-1-a) (A-1-b) (A-1-c)	Impact of cannabis use on fertility Impact of prepregnancy cannabis use on pregnancy and fetus Recommended time between stopping cannabis use and conceiving	Is there a link between cannabis use and infertility? Does a mother’s past cannabis use prior to pregnancy have any impact on a baby in utero? How long should my wife abstain from marijuana before it is safe for her to conceive a child?	21(4.0) 5 (1.0) 1 (0.2)	20 (1.9) 10 (0.9) 6 (0.6)
Total					26 (49.0)	36 (3.4)
Cannabis use during pregnancy						
(B-1)	Safety of cannabis use during pregnancy	(B-1-a) (B-1-b) (B-1-c)	Safety during pregnancy Safety of fetus Withdraw cannabis use for safety consideration	Is smoking weed harmful while pregnant? Is it increasing the risk for the delivery process? I’m 3 weeks pregnant and I didn’t know. So, I ‘ve been drinking and smoking marijuana. Would that impact my baby? What do I do? I am 18 weeks pregnant. If I stop smoking weed, will my baby be okay?	75 (14.2) 238 (45.0) 12 (2.3)	126 (11.7) 451 (42.0) 31 (2.9)
Total					254 (48.0)	483 (45.0)
(B-2)	Recommendations of cannabis use while pregnant	(B-2-a)	Dosing recommendations	How much cannabis per week is safe for pregnant women?	2 (0.4)	3 (0.3)
Total					2 (0.4)	3 (0.3)
(B-3)	Removing cannabis from the body	(B-3-a) (B-3-b)	Time period needed to remove cannabis from the body Method of removal of cannabis from the body	I just found out I’m pregnant. It‘s 7 weeks along. If I stop smoking marijuana now, will it be in my or my baby’s system when it’s born? I’ve been a chronic daily user for about 3 years. What can I do to get weed out of me and the baby’s system before the delivery?	100 (18.9) 32 (6.1)	281 (26.2) 91 (8.5)
Total					127 (24.0)	348 (32.4)
(B-4)	Public opinion on cannabis use while pregnant	(B-4-a)	Public opinion on using cannabis while pregnant	Do you think it is wrong for a pregnant woman to smoke marijuana for nausea, why?	21 (4.0)	35 (3.3)
Total					21 (4.0)	35 (3.3)
(B-5)	Legal consequences of using cannabis while pregnant	(B-5-a) (B-5-b)	Consequences of using cannabis while pregnant Consequences of testing positive while pregnant	Can CPS^a^ take my baby away after birth if I smoked marijuana during my pregnancy, me being 19 years old living in a state where marijuana is legal? I’m 28 weeks pregnant, I stayed with some family over the weekend, and I accidentally ate a edible THC^b^ brownie, thinking it was a regular brownie, What if my son and I test positive for THC in the hospital when he’s born? Will they take my baby away?	45 (8.5) 40 (7.5)	110 (10.2) 65 (6.1)
Total					85 (16.1)	175 (16.3)
(B-6)	Purpose of using cannabis products	(B-6-a) (B-6-b)	Self-reported therapeutic cannabis use: for treating health conditions, pregnancy-associated symptoms, and other health conditions Adult cannabis use	Would there be any circumstance where a doctor would recommend a pregnant woman continue to smoke cigarettes and/or cannabis? This is not about me, I’m not pregnant but I know someone who is claiming this, and I just cannot believe it. Should pregnant women be encouraged to smoke weed if she’s having a complicated pregnancy?	15 (2.8) 7 (1.3)	19 (1.8) 9 (0.8)
Total					21 (4.0)	28 (2.6)

a CPS: Child Protective Services.

b THC: Δ9-tetrahydrocannabinol.

Discussions concerning cannabis use before pregnancy primarily focused on how prior cannabis consumption might affect women who are either already pregnant or preparing for pregnancy (parent code A). We identified user inquiries related to questioning the impact of cannabis use on fertility (A-1-a), exemplified by questions like “could prior cannabis use lead to female infertility?”; impact of prepregnancy cannabis use on pregnancy and the fetus (A-1-b), (eg, “would prior pre-pregnancy cannabis use increase the risk of miscarriage?”), and discussion about the recommended time between stopping cannabis use and conceiving (A-1-c) (eg, “what is the recommended timeframe for ceasing cannabis use before trying to conceive a child?”). Within the cannabis use before pregnancy domain, the subtopics of impact of cannabis use on fertility had the highest volume of questions (A-1-a, 21/529, 4.0% ), followed by the impact of prepregnancy cannabis use on pregnancy and the fetus (A-1-b, 5/529, 1.0% ) and the recommended time between stopping cannabis use and conceiving (A-1-c, 1/529, 0.2%).

Within the domain of cannabis use during pregnancy, the most frequently discussed topics were cannabis safety during pregnancy, detoxing or removing cannabis from the body, and legal consequences associated with cannabis use during pregnancy. Safety-related questions represented the largest subtheme identified in this domain (B-1-[a-c], 314/529, 59.4% ), such as those asking about the potential for cannabis use during pregnancy to trigger a heart attack, (B-1-a, 75/529, 14.2%); about the safety of the fetus, with emphasis on concerns about low birth weight infants (B-1-b, 238/529, 45.0%); and about withdrawal from cannabis use for safety considerations, including users seeking advice on whether they should stop using cannabis and how to do so safely (B-1-c, 21/529, 2.3%). The second-highest volume subtopic included questions about detoxing or removing cannabis from the body (B-3-[a-b], 132/529, 25.0%), such as those asking about the time required (eg, “how can I remove the cannabis out from me and my baby’s body before the birth?”) (B-3-a, 100/529, 18.9%) and methods of detoxing or removal (eg, “how can cannabis be removed quickly?”) (B-3-b, 32/529, 6.1%).

We observed topics related to the legal consequences of using cannabis while pregnant (B-5-[a-b], 85/529, 16.1%), including questions about potential legal consequences of using cannabis while pregnant, (eg, if the pregnant woman would be reported to authorities for using cannabis during pregnancy) (B-5-a, 45/529, 8.5%) and of testing positive while pregnant (eg, concerns about pregnant woman’s baby being taken away due to positive drug test results) (B-5-b, 40/529, 7.6%). Topics related to the purpose of using cannabis products (B-6-[a-b], 22/529, 4.2%) included questions specific to self-reported therapeutic cannabis use (eg, for treating health conditions, pregnancy-associated symptoms, and other health conditions) (B-6-a, 15/529, 2.8%) and adult cannabis use (B-6-b, 7/529, 1.3%). Furthermore, we also detected general topics about public opinion regarding cannabis use while pregnant (B-4-a, 21/529, 4.0%) and recommendations for cannabis use while pregnant (B-2-a, 2/529, 0.4%).

Across all themes, safety-related discussions generated the highest level of user engagement and answer activity (608/1074, 56.6%), followed by detoxing or removing cannabis from the body (B-3, 372/1074, 34.6%) and legal consequences of using cannabis while pregnant (B-5, 175/1074, 16.3%). Across all subtopics, questions related to the safety of the fetus (B-1-b, 451/1074, 42.0%), time period needed to detox or remove cannabis from the body (B-3-a, 281/1074, 26.2%), and the safety of pregnant women (B-1-a, 126/1074, 11.7%) represented the top 3 answer categories in terms of overall user-generated volume.

### Answer Analysis

Using a deductive coding schema, we characterized discussion participants and their attitudes toward cannabis use before and during pregnancy. In total, there were 846 unique users providing 1074 answers. The number of answers per user varied, ranging from 1 (n=722) to 19 (n=1), with a mean of 1.27 (SD 0.99) answers and a median of 1 (IQR 1-1) answer. All answers were reviewed by the first (QX) and second author (CW), coded independently, and achieved high intercoder reliability (Cohen κ=0.91).

User metadata analysis found that among the identified unique Quora users responding to related questions, 5 reported they were pregnant at the time of the post, 86 stated they were formerly pregnant, and 755 were classified as no self-reported information on pregnancy status. Regarding history of cannabis use, 102 self-reported as current cannabis users or past users, 3 reported never using, and 741 did not provide information. Self-reported affiliations included 37 industry observers (ie, members of the cannabis industry), 96 expert observers (eg, health care providers, medical researchers, social work professionals, or similar professionals), 660 nonexpert observers (lacking professional qualifications or affiliations to health care or research), and 53 women indicating cannabis use while pregnant.

Of the 846 Quora users, the majority (n=693, 81.9%) did not disclose their attitudes on general cannabis use. Of those who did express opinions, 9.5% (80/846) conveyed positive, 4.9% (41/846) neutral, and 3.8% (32/846) negative sentiments. A predominant negative sentiment toward cannabis use during pregnancy was observed among a majority of Quora users (524/846, 61.9%), with 3.4% (29/846) individuals expressing positive and 20.7% (175/846) conveying neutral sentiments; 14.0% (118/846) of the Quora users did not express sentiments. Further analysis revealed that of the 32 users demonstrating a negative sentiment toward general cannabis use, 100% (32/32) maintained a negative sentiment toward cannabis use before or during pregnancy. A total of 15 users (15/41, 36.6%) that were initially neutral on general cannabis use shifted to a negative stance on its use during pregnancy. Further, 13.1% (23/176) users exhibited negative sentiments toward pregnancy use but then expressed positive sentiments toward general use. Lastly, 65.7% (455/693) users displayed negative sentiments toward pregnancy use but did not disclose stances on general use.

Results of chi-square testing indicated a significant relationship between Quora users’ attitudes toward cannabis use during pregnancy and their attitudes toward general cannabis use (*χ^2^*_9_=255.73; n=1074; *P*<.001). Individuals with negative views on general cannabis use were also more likely to report negative attitudes toward its use during pregnancy, with a ratio of 100%. In contrast, only 42% (21/50) of users who expressed neutral attitudes on general cannabis use exhibited negative attitude toward use while pregnant. Negative sentiment toward use during pregnancy dropped to 26% (32/122) for those with positive sentiments about general use. These findings suggest a relationship between general attitudes toward cannabis use and perceptions regarding cannabis use during pregnancy.

Among all relevant answers, the major themes included 72.9% (783/1074) answer posts reflecting general opinions about cannabis, 15.0% (161/1074) sharing anecdotes based on personal or observed experiences, 6.0% (64/1074) discussing cannabis-related research, and the remaining 6.0% (64/1074) addressing laws, federal regulations, or recommendations concerning cannabis use in the context of pregnancy.

**Figure 3.**
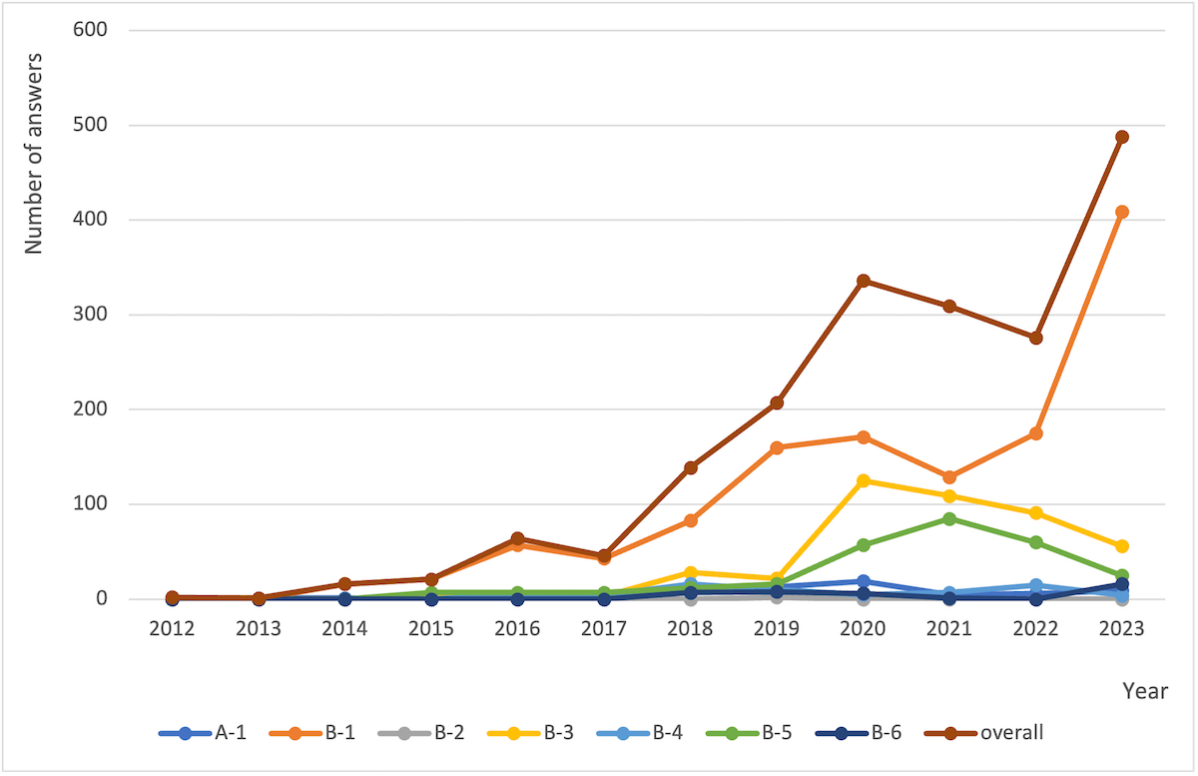
Timeline of Quora answers and corresponding topic themes (2012‐2023). Topic codes and descriptions: A-1-a: impact of cannabis use on fertility; A-1-b: impact of prepregnancy cannabis use on pregnancy and fetus; A-1-c: recommended time between stopping cannabis use and conceiving; B-1-a: safety during pregnancy; B-1-b: safety of fetus; B-1-c: withdraw cannabis use for safety consideration; B-2-a: dosing recommendations; B-3-a: time period needed to remove cannabis from the body; B-3-b: method of removal of cannabis from the body before delivery; B-4-a: public opinion on using cannabis while pregnant; B-5-a: consequences of using cannabis while pregnant; B-5-b: consequences of testing positive while pregnant; B-6-a: self-reported therapeutic cannabis use; B-6-b: adult cannabis use.

After identifying and cross-referencing all relevant answers by the themes of the answers’ associated questions, we used available time stamps (eg, date, mo, and y of answer post) to examine the data from a longitudinal perspective. Based on the volume of answers across themes, we observed a substantial relative increase in discussions related to cannabis and pregnancy over the study period from 2012 to 2023 (Figure 3). Answers to questions specifically associated with the parent topic of safety of cannabis use during pregnancy (B-1) showed a consistent increase in volume over the years observed, with a substantial increase of 93% from 2017 to 2018.

Finally, we conducted analyses on user interaction with answers posted by users. Based on a total of 5585 upvote counts for Quora answers, we noted that answers expressing a negative sentiment regarding cannabis use before or during pregnancy received the highest number of upvotes (n=2687, 48.11%). In contrast, answers conveying sentiments in each other category received fewer upvotes [positive sentiment: 25.25% (1410/5585); neutral sentiment: 21.67% (1210/5585); unknown sentiment: 4.98% (278/5585).

## Discussion

### Principal Findings

This exploratory infodemiology study examining the question-and-answer platform, Quora, analyzed 7834 questions, of which 6.8% (n=529) were identified as relevant to online discussions about attitudes, experiences, and behaviors around cannabis use before and during pregnancy. Our retrospective data mining approach enabled the analysis of a substantial and diverse set of user-generated content (ie, both questions and answers), spanning from July 2012 to July 2023, a time period in which attitudes and policies toward cannabis have rapidly changed in the United States and scientific evidence on the potential harms of cannabis use during pregnancy also grew.

In addition to gathering questions with specific keywords, we included related questions recommended by the Quora platform. This methodology allowed us to capture questions that might have been missed due to our initial keyword selection that may not have included all relevant terms for this topic or the content to which users were exposed. This analysis examined 1916 answers generated from these questions, of which more than half (56.1%, n=1074) were related to the objectives of our study and yielded additional information on user sentiment, interaction, and types of users engaging in this online discourse. Approximately 43.9% (842/1916) of answers were unrelated to the given topic primarily driven by the presence of general smoking-related questions that did not specify whether the response was relevant to tobacco or nicotine smoking or cannabis smoking but nevertheless included user answers for both categories.

Despite changing societal attitudes toward cannabis and the growing accessibility of a variety of cannabis-derived products, online user opinions remain inconsistent about the potential risks associated with use of cannabis by pregnant women, including in the context of users discussing their own past personal experiences, concerns about cannabis use for a current or future pregnancy, perspectives expressed by health care professionals regarding risks, and those from the cannabis industry providing their own opinion about safety. Our analysis of Quora questions uncovered an array of different topics, though most predominantly focused on concerns about the safety of both the pregnant woman and the fetus, and legal implications of perinatal cannabis use. One of the key themes that emerged from the content analysis was the distinction between discussions occurring before and during pregnancy, highlighting the need for better evidence-based information and education about specific risks associated with prenatal and perinatal cannabis use.

Specific detected subthemes focused on the general theme of “safety of cannabis use,” (eg, dosing of cannabis, detoxing or removal, etc), while other questions unrelated to safety (eg, potential legal consequences of cannabis use) were also discussed. While both safety-related and detoxing or removal-related topics generated substantial user engagement, differences in discussion patterns may reflect variation in how users seek and share information regarding cannabis use during pregnancy. However, the heavy skew toward safety content underscores the general risk perceptions among Quora users, who are often looking for answers and advice about cannabis use before and during pregnancy. This finding, evidenced by a high volume of online discussions about safety concerns, aligns with a prior 2021 study examining Twitter posts and similarly detecting a large number of safety-related discussions grouped into periods, including during pregnancy and postpartum, as well as a separate category about cannabis use for pregnancy-related symptoms (not safety related) [41]. While conducting our analysis, we also anticipated encountering conversations or themes related to questions associated with breastfeeding and cannabis use due to documented concerns of safety and general guidance to not use cannabis products while breastfeeding [42]. Hence, it is noteworthy that we did not observe any questions directly addressing breastfeeding and cannabis use despite other studies having shown that risks and benefits are discussed among pregnant women [43]. This absence could be attributed to a lack of specific keywords related to breastfeeding used in study queries.

Examination of answers posted directly by users in response to questions revealed that a minority of users (91/843, 10.7%) specifically identified as being pregnant or formerly pregnant, though the majority did not disclose this information in any manner. Similarly, only 12.1% (102/843) of users explicitly mentioned they were cannabis users, while the majority did not provide information about cannabis use. These findings are not entirely surprising as Quora platform users may be reluctant to fully report their pregnancy status or cannabis use on a public platform, particularly since many Quora users already expressed concerns about legal consequences associated with cannabis use. The participation of various contributors on answer boards, including nonexperts, individuals with personal experiences, and expert observers (eg, health care providers and researchers), as well as cannabis industry observers, adds complexity to the online discourse. Nonexpert observers provided the majority of answers, indicating there may be an imbalance in available evidence-based information sources on the topic, further complicated as health care and public health professionals may offer varying perspectives on the issue or avoid discussion [44,45]. Additionally, Quora is likely not the preferred platform for health care professionals to share detailed, evidence-based information, as they may instead use specialized forums designed for medical discourse (eg, Society of Cannabis Clinicians discussion forums, etc). This contributes to an imbalance in the quality and reliability of information on Quora, as the majority of responses we observed came from nonexpert observers. The observed nonsafety discussions related to legal consequences warrant further analysis. The emergence of these topics suggests that users on Quora are not only concerned about the health implications of perinatal cannabis use but also interested in understanding the legal ramifications that may extend to socioeconomic, social welfare, and parenting considerations. These questions also reflect broader confusion among the public, particularly pregnant women and their families, about the complex and varied landscape of local, state, and federal cannabis laws. This confusion includes uncertainty about the involvement and roles of different authorities (eg, clinicians, social workers, and law enforcement) and the real-world implications of cannabis use, such as the consequences if either the mother or the baby tests positive for cannabis during perinatal care and childbirth. Despite this confusion, many answers did not directly address legality but instead shared general opinions or anecdotal advice and experiences, indicating a need for education and possible legal assistance in this area. Given that Quora may not be a platform where legal experts routinely provide advice, there is likely a notable gap in reliable information around legal topics regarding cannabis use. Professional women’s health societies and other health advocacy groups should consider updating their recommendations, guidelines, and patient education materials to ensure they reflect the most current evidence on cannabis use during pregnancy. Equally important is the provision of comprehensive education and resources to health care providers so they can effectively counsel patients on this matter. If health care providers are empowered with the necessary tools and knowledge, patients may be more likely to seek advice from qualified professionals rather than relying on sources like social media for medical guidance.

Finally, longitudinal analyses of answers posted by users allowed us to observe changes in discussions and in the types of questions that have become more predominant as attitudes and state-level policies towards cannabis use have become more permissive. Overall, the volume of conversations associated with cannabis and pregnancy has increased, likely due to increased use of cannabis in the general population, necessitating the need for convenient and accessible information sources, such as online platforms, on cannabis safety and its potential legal implications. The subtopic category that saw the highest rate of increase was safety of cannabis use during pregnancy, further emphasizing the need for better evidence, dissemination, and targeted health communication so that women can better understand the potential risks associated with cannabis use during this critical time of human development. Specifically, the increase in safety-related discussions observed between 2017 and 2018 may reflect broader changes in cannabis legalization and increased public acceptance and interest in cannabis products and their safety. For context, nearly 50 cannabis-related bills were introduced in Congress, and California began legal state-licensed retail sales of adult use cannabis during this time period [46,47].

### Limitations

The study is exploratory and has limitations inherent to analyzing user-generated content on social media platforms. The keywords used for data collection may not comprehensively cover all aspects related to pregnancy and cannabis use. This limitation may result in missing relevant discussions or perspectives that use different terminology or phrases not included in the selected keywords. Additionally, user classifications were based on publicly available self-disclosed profile information, and some users may have possessed relevant expertise without explicitly reporting professional credentials or affiliations on their Quora profiles. The study focused solely on analyzing questions and answers and did not examine user-generated Quora comments on answers. The omission of comments may result in the unintended consequence of overlooking additional insights, nuances, or counterarguments presented in the discussion threads. Additionally, the online nature of the discussions may not fully capture the nuances of face-to-face conversations or the diversity of perspectives particularly among pregnant women who do not engage in online conversations or information seeking.

### Conclusions

The results from this study provide insight into the attitudes, experiences, behaviors, and sentiments surrounding cannabis use during pregnancy in the context of online discourse occurring on a popular question-and-answer social media platform. The themes generated from this study provide new insights into observed gaps in online discussions and information-sharing patterns regarding the potential harms of cannabis use during pregnancy, while also highlighting the importance of evidence-based communication and public health outreach. These findings highlight how Quora users discuss cannabis use during pregnancy, including themes related to safety perceptions, anecdotal experiences, legal concerns, and information-seeking behaviors. Future studies should continue exploring online attitudes and perceptions regarding cannabis use during pregnancy with additional data sources and infodemiology approaches.

## Abbreviations

IRB: Institutional Review Board
THC: Δ^9^-tetrahydrocannabinol
